# Thermal Properties of Plasticized Cellulose Acetate and Its β-Relaxation Phenomenon

**DOI:** 10.3390/polym13091356

**Published:** 2021-04-21

**Authors:** Rafael Erdmann, Stephan Kabasci, Hans-Peter Heim

**Affiliations:** 1 Institute for Biopolymers and Sustainability (ibp), University of Applied Science Hof, Alfons-Goppel-Platz 1, 95028 Hof, Germany; 2 Institute of Materials Engineering, University of Kassel, Mönchebergstraße 3, 34125 Kassel, Germany; Heim@uni-kassel.de; 3 Department Circular and Bio-based Plastics, Fraunhofer UMSICHT, Fraunhofer Institute for Environmental, Safety, and Energy Technology, Osterfelder Str. 3, 46047 Oberhausen, Germany; Stephan.Kabasci@umsicht.fraunhofer.de

**Keywords:** cellulose acetate, plasticized cellulose acetate, bio-based polymers, glass temperature depression, plasticizer, glycerol triacetate, triethyl citrate, beta relaxation, activation energy

## Abstract

Cellulose acetate (CA), an organic ester, is a biobased polymer which exhibits good mechanical properties (e.g., high Young’s modulus and tensile strength). In recent decades, there has been significant work done to verify the thermal and thermomechanical behaviors of raw and plasticized cellulose acetate. In this study, the thermomechanical properties of plasticized cellulose acetate—especially its β-relaxation and activation energy—were investigated. The general thermal behavior was analyzed and compared with theoretical models. The study’s findings could be of special interest, due to the known β-relaxation dependency of some polymers regarding mechanical properties—which could also be the case for cellulose acetate. However, this would require further investigation. The concentration of the plasticizers—glycerol triacetate (GTA) and triethyl citrate (TEC)—used in CA ranged from 15 to 40 wt%. DMTA measurements at varying frequencies were performed, and the activation energies of each relaxation were assessed. Increasing plasticizer content first led to a shift in β-relaxation temperature to highervalues, then reached a maximum before declining again at higher concentrations. Furthermore, the activation energy of the β-relaxation constantly rose with increases in plasticizer content. The trend in the β-relaxation temperature of the plasticized CA could be interpreted as a change in the predominant phase of the overlapping β-relaxation of the CA itself and the $\alpha^{\prime }$-relaxation of the plasticizer—which appears in the same temperature range. The plasticizer used (GTA) demonstrated a higher plasticization efficiency than TEC. The efficiencies of both plasticizers declined with increasing plasticizer content. Additionally, both plasticizers hit the saturation point (in CA) at the lowest studied concentration (15 wt%).

## 1. Introduction

Cellulose acetate (CA), an organic ester, is a biobased polymer which exhibits good mechanical properties (e.g., high Young´s modulus and tensile strength). Its general properties can be tailored by the addition of low molecular plasticizers, which are also necessary for processing. In recent decades, different types of plasticizers have been incorporated in CA, based on glycols [1,2], phthalates [3,4,5], acetates [4] and citrates [2,6]. Their compatibility—as well as the resulting mechanical and thermal properties of plasticized CA—have been analyzed. The most efficient known plasticizers for cellulose acetate are: diethyl phthalate (DEP), glycerol triacetate (GTA) and triethyl citrate (TEC).

Comprehensive data are available on how plasticizers influence the mechanical properties of cellulose acetate [3,6,7,8]. Plasticizers can have a tremendous impact on the thermal and thermomechanical properties of CA, depending on the type and concentration used.

Cellulose acetate itself possesses a narrow temperature window between its glass transition temperature $T_{g}$ and its degradation temperature $T_{d}$ [9]. Plasticizers are used to broaden this processing window and soften the material. This method is state-of-the-art, but has a negative effect; it diminishes the product service temperature, due to the reduction in glass transition temperature. In this study, two different plasticizers (glycerol triacetate (GTA) and triethyl citrate (TEC)) were used. Their efficiency at reducing the glass transition temperature of CA was analyzed and compared with existing theoretical models—specifically, the Fox, Kelley–Bueche and Couchman–Karasz models [10,11,12]. Furthermore, the α- and β-relaxation of CA were examined, as were its interactions with the plasticizers used in the study. It is well known that β-relaxations can have a high impact on the mechanical properties of various polymers. Therefore, this survey focused on the β-relaxation temperature and its shifts, and how they affect the product service temperature of CA. The relaxation intensities and shifts in tanδ of CA depended highly on the plasticizer type and concentration. In the scientific community, the β- and γ-relaxation of unplasticized cellulose acetate are not fully understood; they are still a controversial discussion topic [13]. The β-relaxation of unplasticized cellulose acetate could be due to the cooperative motion of the side groups with the main chain—or motions of single monomeric units. Different opinions on the γ-relaxation of cellulose-based materials were summarized by Einfeldt et al. [13]. The first interpretation of γ-relaxation described it by suggesting that only the methyl side group of the glucopyranose unit [14,15,16,17] could freely rotate. Others proposed that both side groups (methyl as well as hydroxyl) could freely rotate [18,19,20]. Another reasonable explanation suggested that local motions of the cellulose chain under a thermodynamic point of view were comparable with β-relaxation motions [21,22,23]. McBrierty et al. and others justified γ-relaxation with bounded water [24,25]. The final summarized interpretation of γ-relaxation was based on an energetic point of view, wherein an individual glucopyranose unit changed from boat to chair conformation [23].

This paper presents results of an investigation of the thermal properties of cellulose acetate and the activation energies of temperature-dependent transitions. DMTA measurements were performed with different plasticizer types and concentrations. General thermal behavior—i.e., glass transition temperature and its reduction—was analyzed and compared with theoretical models.

## 2. Materials and Methods

### 2.1. Materials

Cellulose acetate (CA) powder (CS-Grade) was purchased from Sichuan Push Acetati Co., Ltd., Changning, CN, with an acetylation content of approximately 40%, which refers to a degree of substitution (DS) of ~2.5. The average particle size (d_50_) of the powder was 250 μm and its specific gravity was in the range of 1.20–1.32 g⋅cm^−3^. The glass transition temperature was approximately 198 °C and the melting temperature was within the range of 230–250 °C.

The plasticizers used were: glycerol triacetate (GTA) and triethyl citrate (TEC). Table 1 shows the characteristics of the materials taken from the technical and material safety data sheets of the manufacturers. GTA (EDENOR^®^ GTA), as a colorless liquid, was kindly provided by KLK Emmerich GmbH, Düsseldorf, DE. TEC (CITROFOL^®^ AI), another liquid plasticizer, was purchased from Jungbunzlauer Deutschland GmbH, Ladenburg, DE. The materials were used as received.

### 2.2. Sample Preparation

**Figure 1:**
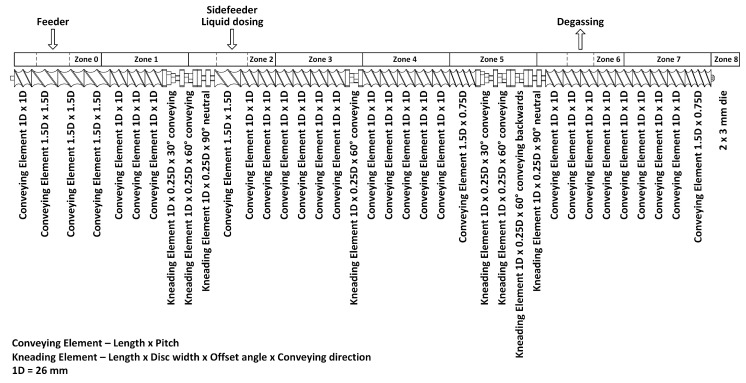
Screw design for compounding plasticized cellulose acetate (CA).

Cellulose acetate compounds with varying plasticizer contents were prepared in a co-rotating intermeshing twin-screw extruder EMP 2640, TSA Industriale S.r.l., Cernobbio, IT, with a screw diameter of 26 mm and an L/D ratio of 40. A special screw design (with a low amount of shear/kneading elements) was used for processing (Figure 1). The throughput was kept constant at 10 kg h^−1^, and the screw speed was set at 250 rpm. The temperature profile of the extruder was set from the feeding zone (zone 0) to the die (zone 8) as follows: 100 °C; 162 °C; 167 °C; 173 °C; 173 °C; 180 °C; 185 °C; 204 °C; and 210 °C. The liquid plasticizers were added via a peristaltic pump (volumetric feeding) in zone 2. Cellulose acetate (as a very hydrophilic polymer) was dried overnight at 80 °C in a dry-air dryer prior to processing. The dry cellulose acetate powder was fed over the main hopper. The melt was extruded through a dual strand die with a diameter of d_0_ = 3 mm before being cooled in a water bath and pelletized.

After extrusion, the compounds were injection molded with a Battenfeld BA 600/125 CDC, Wittmann Battenfeld Deutschland GmbH, Nürnberg, DE. The injection unit 125 was equipped with a core-progressive screw with a diameter of 25 mm and an L/D ratio of 21.6. It was capable of applying a maximum specific injection pressure of 2149 bar. The injection unit had an open nozzle of d_0_ = 3 mm and a maximum melt capacity of 58.9 cm^3^. The clamping force was adjustable up to 600 kN. A slide-in tool (tempered at 40 °C, with a specific geometry for producing test specimens according to the UL94 norm) was used to produce the samples. The plasticized cellulose acetate was dried for four hours at 60 °C in a dry-air dryer prior to processing. The temperature profile of the injection unit for the compound with a lower amount of plasticizer (15–25 wt%) was set from the feeding zone (zone 1) to the nozzle (zone 4) as follows: 200 °C; 205 °C; 210 °C; and 220 °C. The temperature profile of the CA compound with a higher amount of plasticizer (30–40 wt%) was adjusted (due to the better flowability of the melt) to: 185 °C; 190 °C; 195 °C; and 200 °C. The volumetric flow rate was set at 30 cm^3^⋅s^−1^. The resulting specific injection pressure varied between ~780–1550 bar, depending on plasticizer concentration. The holding pressure was set at 600 and 1000 bar for compounds with higher (30–40 wt%) and lower (15–25 wt%) amounts of plasticizer, respectively, and pressure was held for 15 s each.

The injection-molded specimens (measuring 126 mm × 13 mm × 1.7 mm) were cut into the required sample size (35 mm × 13 mm × 1.7 mm) for DMTA measurement. The samples were kept in a desiccator to avoid water absorption prior to testing. For DSC measurements, the plasticized granulates (compounds) from the twin-screw extrusion process were used.

### 2.3. Characterization and Sample Preparation

#### 2.3.1. Differential Scanning Calorimetry

To analyze the phase transitions of the materials, differential scanning calorimetry (DSC) was performed with the DSC 204 F1 Phoenix^®^, Erich NETZSCH GmbH & Co. Holding KG, Selb, DE, which was equipped with a liquid nitrogen cooling system. The calibration of the device was done to the following standards: adamantane, n-dodecane, n-octadecane, indium, bismuth, and zinc. The samples (CA powder and granules), which had an initial weight of 10 mg, were placed in an aluminum pan with a pierced lid. The measurements were performed with a heating and cooling rate of 20 K⋅min^−1^ under nitrogen atmosphere (purge and protective gas 20 mL⋅min^−1^) to avoid oxidation reactions. The samples were cooled down to −100 °C, heated up to 250 °C, cooled again to −100 °C and reheated up to 250 °C. Between the heating and cooling periods, the samples were kept constant under isothermal conditions at maximum low and high temperatures (for 15 min at −100 °C and 5 min at 250 °C), respectively. The phase transitions of the materials were analyzed via NETZSCH Proteus^®^ 7.1 software, Erich NETZSCH GmbH & Co. Holding KG, Selb, DE.

To give a qualitative theoretical prediction for the glass transition reduction of plasticized cellulose acetate with increasing plasticizer content, three theoretical models were applied. These models were correlated with the experimental data determined from DSC measurements. The first was the Fox model, wherein the resulting glass transition temperature of the compound depends on the glass transition temperature and the mass fraction of each single component.

The glass transition temperature of the compound $T_{g^{\mathrm{Comp}.}}^{F}$ can be calculated according to the Fox model [10] with the Equation (1):

(1) $$\frac{1}{T_{g^{\mathrm{Comp}.}}^{F}}=\frac{\omega_{\mathrm{CA}}}{T_{g^{\mathrm{CA}}}}+\frac{\omega_{\mathrm{PL}.}}{T_{g^{\mathrm{PL}.}}}$$

where $\omega_{\mathrm{CA}}$ and $\omega_{\mathrm{PL}.}$ are the mass fractions of cellulose acetate and the plasticizer, and $T_{g^{\mathrm{CA}}}$ and $T_{g^{\mathrm{PL}.}}$ are the corresponding glass transition or pour point temperatures of cellulose acetate and the used plasticizer.

Another approach used to fit the experimental data was the Kelley–Bueche [11] or Gordon–Taylor model [26]. They expanded the Fox equation with the factor k which represents the free volume ratio of the two components, cellulose acetate and plasticizer.

(2) $$T_{g^{\mathrm{Comp}.}}^{\mathrm{KB}}=\frac{\left(\omega_{\mathrm{CA}}\cdot T_{g^{\mathrm{CA}}}+k\cdot \omega_{\mathrm{PL}.}\cdot T_{g^{\mathrm{PL}.}}\right)}{\left(\omega_{\mathrm{CA}}+k\cdot \omega_{\mathrm{PL}.}\right)}$$

The abbreviations of the Kelley–Bueche or Gordon–Taylor model (Equation (2)) are the same as in Equation (1).

(3) $$k=\frac{\rho_{\mathrm{CA}}\cdot ∆\alpha_{\mathrm{PL}.}}{\rho_{\mathrm{PL}.}\cdot ∆\alpha_{\mathrm{CA}}}=\frac{\rho_{\mathrm{CA}}\cdot T_{g^{\mathrm{CA}}}}{\rho_{\mathrm{PL}.}{\;T}_{g^{\mathrm{PL}.}}}$$

The constant k can be estimated from the density ratio between the polymer $\rho_{\mathrm{CA}}$ and the plasticizer $\rho_{\mathrm{PL}.}$ and the corresponding slopes of the expansion coefficients of the polymer $∆\alpha_{\mathrm{CA}}$ and the plasticizer $∆\alpha_{\mathrm{PL}.}$ near their glass transition temperatures. The factor k can be calculated using the densities and the glass transition temperatures, applying the simplified Sigma–Boyer Equation ($∆\alpha \cdot T_{g}\sim \mathrm{constant})$, cf. Equation (3) [27]. Furthermore, k is often used as a fit parameter to estimate the interaction intensities between the polymer and the plasticizer. High values of k indicate low interactions between the two components, while low values point at high interactions.

The third, the Couchman–Karasz model, is based on the entropy continuity of the mixture at $T_{g}$ [12]. If it is assumed that $∆C_{P}$ is inversely proportional to temperature, then the following Equation (4) is derived [28].

(4) $$T_{g^{\mathrm{Comp}.}}^{\mathrm{CK}}=\frac{\omega_{\mathrm{PL}.}\cdot {\Delta C}_{p^{\mathrm{PL}.}}\cdot T_{g^{\mathrm{PL}.}}+\omega_{\mathrm{CA}}\cdot {\Delta C}_{p^{\mathrm{CA}}}\cdot T_{g^{\mathrm{CA}}}\;}{\omega_{\mathrm{PL}.}\cdot {\Delta C}_{p^{\mathrm{PL}.}}+\omega_{\mathrm{CA}}\cdot {\Delta C}_{p^{\mathrm{CA}}}}$$

The abbreviations are the same as in the Equations (1) and (2) except the heat capacities of the cellulose acetate ${\Delta C}_{p^{\mathrm{CA}}}$ and the used plasticizer ${\Delta C}_{p^{\mathrm{PL}.}}$.

To analyze the glass temperature depression onto cellulose acetate in dependence of the plasticizer concentration, the Equation (5) can be applied.

(5) $$∆T_{g}=T_{g^{\mathrm{CA}}}-T_{g^{\mathrm{pCA}}}$$

The glass temperature depression $∆T_{g}$ depends on the relation between the glass transition temperatures of the raw $T_{g^{\mathrm{CA}}}$ and plasticized $T_{g^{\mathrm{pCA}}}$ cellulose acetate.

#### 2.3.2. Dynamic Mechanical Thermal Analysis

Dynamic mechanical thermal analyses (DMTA) were performed with a Modular Compact Rheometer MCR 302, Anton Paar Germany GmbH, Ostfildern, DE, equipped with a clamping unit SRF 10 for rectangular-shaped samples. A convectional heating device CTD 600—with a control unit TC 30—was used to heat the samples, and a liquid nitrogen evaporating unit EVU 20 was used to cool them. The measurements were performed with a heating rate of 3 K⋅min^−1^ in a nitrogen atmosphere under torsion with a deformation rate of 0.01% and a frequency of 1 Hz. The samples were cooled down to −150 °C and then heated up to 160 °C. To determine the characteristic values of the samples, Origin^®^ 2019 software, OriginLab Corporation, Northampton, MA, USA, was used. To calculate the activation energy for every degree of relaxation (α-, β- and γ-relaxation), additional measurements at different frequencies of 10, 30, and 50 Hz were performed. The activation energy [29] can be calculated via the Equation (6):

(6) $$\ln\left(\frac{f}{f_{\mathrm{ref}}}\right)=-\frac{E_{a}}{R\cdot T_{\mathrm{rel}.}}$$

where $E_{a}$ is the activation energy, f and $f_{\mathrm{ref}}$ are the applied and reference frequency (1 Hz), respectively, $T_{\mathrm{rel}.}$ is the specific relaxation temperature of each degree of relaxation and R is the universal gas constant.

## 3. Results and Discussion

**Figure 2:**
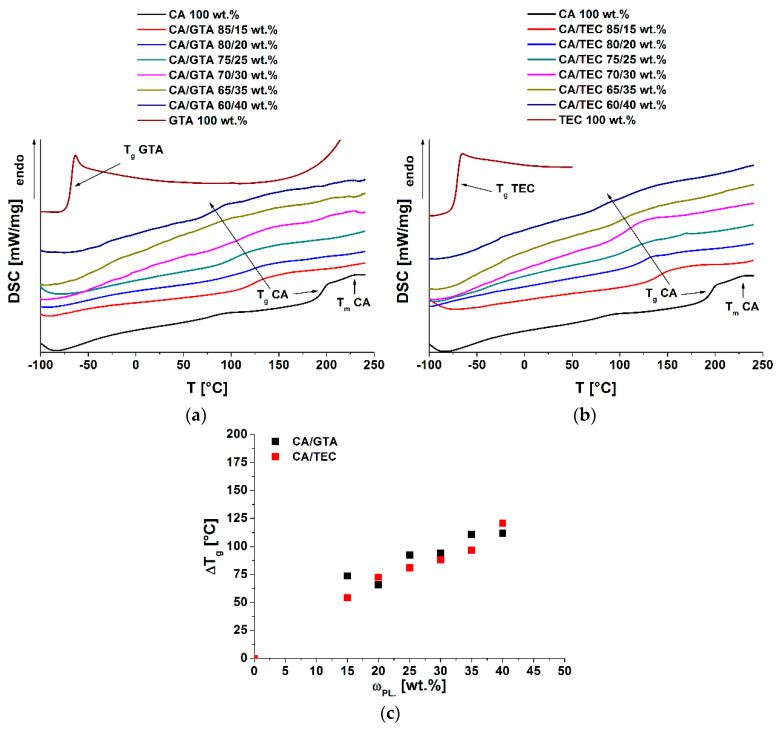
Second heating run of the corresponding samples (**a**) samples with GTA and (**b**) samples with TEC as plasticizer in cellulose acetate. (**c**) Glass transition temperature depression of GTA- and TEC-plasticized CA.

In this work, two different plasticizers (GTA and TEC) were used to analyze the thermal behavior of cellulose acetate in dependence of plasticizer type and concentrations. To determine the glass transition temperature and its depression, DSC measurements were performed. Figure 2 shows the second heating run of the corresponding samples.

The raw cellulose acetate powder showed a high glass transition temperature near to its melting temperature. A low melting enthalpy was visible, as is known for cellulose esters [30,31,32]. Furthermore, cellulose acetate with ${\Delta C}_{p^{\mathrm{CA}}}$ = 0.233 J g^−1^⋅K^−1^ exhibited a very low heat capacity change in comparison to the plasticizers GTA (${\Delta C}_{p^{\mathrm{GTA}}}$ = 0.777 J g^−1^⋅K^−1^) and TEC (${\Delta C}_{p^{\mathrm{TEC}}}$ = 0.890 J g^−1^⋅K^−1^), as shown in Figure 2. With the addition of plasticizer, no crystallization of CA was observed. The pour points of the pure plasticizers GTA and TEC were visible in DSC measurements. However, the plasticizer pour points in the compounds could not clearly be detected by DSC measurements under the used conditions because they formed homogeneous mixtures with CA. The plasticizers GTA and TEC had a significant influence on the glass transition temperature of cellulose acetate [33]; both decreased the glass transition temperature of CA significantly, although GTA seemed to be more effective. This could be determined by the slightly higher glass transition temperature depression of GTA in comparison to TEC (Figure 2c, Table 2). Guo et al. used GTA to alter the permeability and mechanical properties of cellulose acetate films. They also confirmed the high effectiveness of GTA [1]. The difference in the glass transition temperature depression of plasticized cellulose acetates may be caused by the interactions—hydrogen bonds and dipole-dipole interactions—between the plasticizer and cellulose acetate itself. Stickney et al. determined that the plasticization was very complex and depended on a variety of criteria [34], e.g., the amount, type, and accessibility of the functional groups in the plasticizer and the polymer chain. Cellulose acetate contains a high amount of free hydroxyl groups, depending on its degree of substitution. Triethyl citrate also contains a free hydroxyl group which can interact with CA, in contrast with GTA. Glycerol triacetate acts more as a shielding plasticizer, and thus reduces the intermolecular forces between the CA chains. In comparison to TEC, this leads to a higher glass transition temperature depression.

The glass transition depressions of the plasticized CA were analyzed in more detail. The theoretical values—according to the Fox, Kelly–Bueche and Couchman–Karasz models—were determined. (Equations (1), (2) and (4)).

**Figure 3:**
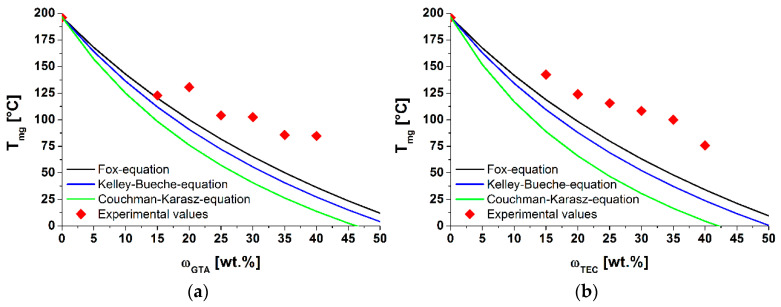
Experimental and theoretical values of the glass transition temperature for (**a**) GTA- (k=2.66) and (**b**) TEC (k=2.74) -plasticized CA.

As shown in Figure 3, the theoretical models did not correctly predict the experimental values of the $T_{g}$ of GTA- and TEC-plasticized CA within the studied concentration range. A higher deviation from the theoretical calculated values was observed for the TEC-plasticized CA. As mentioned, GTA is a very effective plasticizer. Therefore, the glass transition temperature depressions at lower plasticizer concentrations were higher than observed for TEC. At higher plasticizer concentrations (>25 wt%), the plasticizer effectiveness of GTA decreased, while the glass transition temperatures of both compounds were equal at the highest plasticizer concentration (40 wt%). Bao analyzed the glass transition temperature of DEP- and GTA-plasticized CA in concentrations ranging up to 50 wt% via modulated DSC (MDSC) [35]. He found only one $T_{g}$ for CA films containing less than 20 wt% GTA. For CA films with a higher amount of plasticizer, two $T_{g}$´s were evident. This indicated a starting phase separation of the two components. In Figure 2a, no glass transition temperatures of GTA itself were visible in any of the compounds, but we confirmed the deviation of the theoretical values with plasticizer contents larger than 15 wt%. These phenomena also seemed to occur for TEC-plasticized CA. Here, the phase separation—what we interpreted as a saturation point—took place earlier. Similar findings were also observed by Scandola and Ceccorulli for diethyl phthalate (DEP)-plasticized CA [14,36]. Their theoretical predictions only fit the experimental data up to 15 wt% DEP. Bao et al. confirmed these findings [5]. In addition, they were able to verify a starting phase separation at plasticizer contents exceeding 25 wt% of DEP, due to the appearing glass transition temperature of the plasticizer itself. In addition, Bao detected a starting phase separation for the plasticizer GTA in cellulose acetate. In comparison to the plasticizer DEP, the phase separation began as the concentration of 30 wt% (GTA in CA) was reached. In all of our DSC measurements, no second $T_{g}$ ($T_{g}$ of the GTA itself)—which would have indicated the starting phase separation—could be detected. Likewise, no phase separation could be seen in SEM images (Appendix A), as was shown by Guo et al. for PEG-plasticized CA compounds [37].

**Figure 4:**
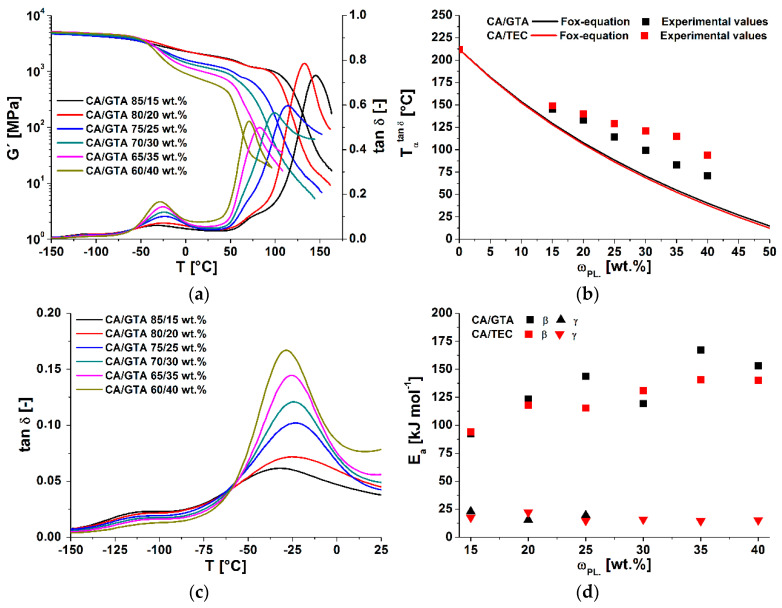
Results of the DMTA measurements of different plasticized cellulose acetate. (**a**) tanδ of GTA and TEC plasticized CA; (**b**) experimental and theoretical values of the α -relaxation temperature; (**c**) tanδ of β- and γ-relaxation temperature; (**d**) activation energies of the β- and γ-relaxation of GTA and TEC plasticized CA.

To analyze the effects of plasticizer type and content on the main relaxation processes in cellulose acetate (α-, β- and γ-relaxation), dynamic mechanical analyses at different frequencies were performed. The tanδ and storage modulus $G^{\prime }$ of plasticized cellulose acetate with different contents of GTA, measured at 1 Hz, are shown in Figure 4a. The α-relaxation at $T_{\alpha }^{\tan\delta }$ corresponded to the glass transition temperature $T_{g}$. It shifted with increasing amounts of plasticizer to lower temperatures, due to the plasticization effect (increase in free volume) [38]. Furthermore, the intensities of tanδ of the α-relaxation declined with increasing plasticizer content, as did the storage moduli at the beginning of the glass transitions. Both effects are well known and comprehensive data for different plasticizers in CA are available [4,5,14]. In Figure 4b, the measured data were compared with the additivity model of Fox. Due to sample preparation issues, no DMTA data for the raw cellulose acetate could be determined. The value was taken from the literature [5]. The glass transition temperatures of the plasticized cellulose acetate deviated from the theoretical model in the examined concentration range. These deviations can be explained by different factors: first, the Fox model is an empirical model and is normally used for polymer-diluent systems. It does not account for hydrogen interactions, which predominate in cellulose derivatives. Seymour et al. noted that plasticizers have less influence on the $T_{g}$ of cellulose derivatives than was predicted by different models [39]. This discrepancy increased with greater amounts of plasticizer. Furthermore, Scandola and Ceccorulli stated that small amounts of plasticizer solvated the amorphous phase of cellulose acetate [14]. Higher amounts can penetrate the crystalline fractions, which are 9–16% less present in cellulose diacetate (DS = 2.5) [5]. These observations were confirmed on triacetin-plasticized cellulose acetate by Bao. As mentioned in the first section, the main deviation from the Fox model was due to the starting phase separation, which was confirmed for GTA- and DEP-plasticized CA systems via MDSC [5,35]. Gloor et al. noted that the miscibility of cellulose esters declined with increasing amounts of C-atoms in the side chain of the plasticizer [40]. In comparison to GTA, triethyl citrate has three methyl groups and one hydroxyl group. Therefore, the start of the phase separation at a lower plasticizer content of TEC (in comparison to GTA) could be possible. However, as was deduced from DSC results, our DMTA measurement did not reveal any evidence of phase separation of GTA and TEC in plasticized cellulose acetate.

The β-relaxation processes of CA and plasticized CA are not fully understood, and are still a controversial topic [5,14,39]. First, increasing GTA content led to a shift in the β-relaxation to higher temperatures. Then, the transition temperature $T_{\beta }^{\tan\delta }$ reached a maximum, and afterwards declined again, as shown by magnification in Figure 4c and the derived values in Table 3. A different trend was seen with TEC as plasticizing agent. Here, the β-relaxation temperature initially increased, but then remained constant at plasticizer contents of more than 30 wt% (see Table 3). In the literature, it has been described that—with increasing plasticizer content—the β-relaxation temperature shifts to lower values. The preconditions for this behavior are that the glass transition (e.g., pour point temperature) of the plasticizer is lower than the β-relaxation temperature of cellulose acetate. Unplasticized cellulose acetate shows a β-relaxation temperature of −20 °C [39]. The first increase of the β-relaxation temperature of TEC- and GTA-plasticized cellulose acetate was also seen in different plasticized cellulose acetate systems [14,35].

In addition to the investigations at 1 Hz (shown in Figure 4), further DMTA measurements at frequencies of 10, 30 and 50 Hz were performed. With increasing frequency, the peak maxima of all relaxation processes (α-, β- and γ-relaxation) shifted to higher temperatures. The activation energies of the β-relaxation were assessed according to Equation (6). With greater plasticizer concentration, the activation energy of the β-relaxation still rose (see Figure 4d). The β-relaxation activation energy of unplasticized cellulose acetate is given in the literature as 76–80 kJ⋅mol^−1^ by Sousa et al. [41] and ~85 kJ⋅mol^−1^ by Montes et al. [42]. Extrapolating our data to zero plasticizer content yielded values in the same range. A possible explanation for the increase of the activation energy was given by Scandolla and Ceccorulli [14]. They suggested that, in addition to the interactions of the plasticizers with cellulose acetate (hydroxyl interactions), weaker interactions between the plasticizer and the acetate-groups of the glucopyranose ring are formed. These interactions could lead to an increase in the size of the units which are responsible for the β-relaxation phenomenon of CA. From their experiments with DEP as plasticizer, they found that the activation energy of the β-relaxation was constant up to 15 wt% concentration. With further increases, the activation energy rose linearly, up to 222 kJ⋅mol^−1^ at a plasticizer concentration of 50 wt%. An increase of the activation energy of DEP-plasticized CA was also reported by Bao et al. in a concentration range of 15 to 45 wt% [5]. An additional explanation that supported the findings of Scandolla and Ceccorulli [14] was given by Einfeldt et al. [13]. They analyzed the influence of water on the dielectric properties of cellulose. They concluded that water solvated the hydrophilic groups of the glucopyranose unit, and that these water molecules increased the dipole moment and the moment of inertia of the movable groups [13]. Furthermore, the water molecules build a bridge parallel to the glycosidic linkage along the main chain, which led to a higher stiffness of the main chain. It was also reported that higher ordered structures of biopolymers were more stable in wet states. The increase in activation energy as water content increased could be attributed to the stabilizing effect of the main chain. Contrarily, the macroscopic flexibility was increased by the swelling power of water. These phenomena, explained by Einfeldt et al. [13], could also be the case for other swelling agents like plasticizers.

Different explanations have been reported to explain the γ-relaxation movements of cellulose acetate. The most supported explanation is local motion of the -CH_2_-OH and –OH groups of one pyranose unit [43]. Sousa et al. proposed activation energies of 32 and 52 kJ⋅mol^−1^ for the γ-relaxation [41]. Another possible explanation for the γ-relaxation of amorphous cellulose was given by the computer simulations of Montes et al. [42]. They concluded that the energy barrier for a rotation of the glucose ring around a single glycosidic linkage β (1–4) for the γ-relaxation were in the order of 20 kJ⋅mol^−1^. Triethyl-citrate-plasticized cellulose acetate showed γ-relaxation peaks over the whole concentration range. The γ-relaxation peaks of GTA-plasticized CA instead declined with increasing plasticizer content—and vanished with increasing frequency. Therefore, no activation energies could be determined for the compounds with concentrations of GTA over 25 wt%. The assessed γ-relaxation activation energies for GTA- and TEC-plasticized CA were approximately constant at ~20 kJ⋅mol^−1^ (Table 3) over the studied concentration range. In comparison to other plasticized cellulose acetate systems, our values were very low, but they fit the findings of Montes et al. [42].

## 4. Conclusions

The thermal and thermomechanical properties of plasticized cellulose acetate—in dependence of plasticized type and concentration—were investigated. The plasticizers glycerol triacetate (GTA) and triethyl citrate (TEC) were used in concentrations ranging between 15 wt% and 40 wt%. To predict the glass transition temperature of plasticized CA, the theoretical models proposed by Fox, Kelley–Bueche and Couchman–Karasz were applied. Values from these equations were compared with experimental data determined by DSC measurements. The theoretical models overestimated glass transition temperature reductions in the studied concentration range. The glass transition temperature of plasticized CA constantly declined with increasing plasticizer content. Glycerol triacetate seemed to be more suitable than TEC for plasticization of cellulose acetate, due to the higher glass transition temperature depression.

DMTA measurements were conducted to analyze the relaxation behavior of the plasticized cellulose acetate. Furthermore, the activation energies of the β- and γ-relaxation were assessed. Increasing plasticizer contents led to a shift of the α-relaxation to lower temperatures and a decline in the intensities of the tanδ peaks. The same behavior (a greater glass temperature reduction in GTA-plasticized CA) was observed in DSC measurements. A different behavior for the two plasticizers was observed for the β- and γ-relaxations. The β-relaxation temperature of GTA-plasticized CA first increased, then reached a maximum, and then declined again with increasing GTA content. The intensity increased constantly. A different behavior was observed for TEC-plasticized CA. Here, the β-relaxation temperature shifted to higher temperatures up to 25 wt% concentration. Above that value, the temperature remained constant. These temperature shifts cannot currently be explained. The peak maxima of the γ-relaxation remained at the same temperature, and their intensities declined. However, the increase of the β-relaxation activation energy as plasticizer content increased could be explained by the suggestions of Scandola and Ceccorulli, and the findings of Einfeldt et al. They suggest that, in addition to the main interactions (hydroxyl interactions), weaker interactions between the plasticizer and the acetate-groups of the glucopyranose ring occur. These interactions lead to an increase in the size of the unit, which is responsible for the β-relaxation phenomenon—and consequently to the increase in activation energy. Additionally, the plasticizer molecules could build a bridge parallel to the glycosidic linkage along the main chain, which would lead to a higher stiffness in the main chain dipole moment and the moment of inertia of the movable groups, as has been described for wet cellulose. The activation energies of the γ-relaxations of both plasticizers remained constant in the studied concentration range.

Further and more precise investigations need to be performed to analyze the unusual behavior of the β-relaxation temperature shifts of plasticized cellulose acetate.

In conclusion: cellulose acetate (CA), as an organic ester, is a biobased polymer which exhibits good mechanical properties (e.g., a high Young´s modulus and tensile strength). Depending on the degree of substitution, CA is compostable—and plasticized CA could replace materials like PP, PS or ABS in injection-molded applications as an environmentally friendly polymer.

## Figures and Tables

**Table 1:** Characteristic data of the plasticizers used.

Plasticizer	ρ	Acidity	$\eta_{dyn.}$	**APHA**	**NTU**	**n**	$T_{p}$	$T_{b}$
	[g cm^−3^]	[%]	[mPa⋅s]	**[-]**	**[-]**	**[-]**	**[°C]**	**[°C]**
GTA	1.157–1.159 ^1^	0.045	21–24 ^1^	15	-	1.4307–1.4319 ^1^	−78	258–259
TEC	1.135–1.139 ^2^	0.02	35.2 ^2^	30	2	1.439–1.441 ^2^	−45.5	127 ^3^

ρ density; $\eta_{dyn.}$ dynamic viscosity; APHA (American Public Health Association) color scale; NTU (nephelometric turbidity unit) turbidity; n refractive index; $T_{p}$ pour point; $T_{b}$ boiling point. ^1^—properties measured at 20 °C. ^2^—properties measured at 25 °C. ^3^—property measured at a pressure of 1.33 mbar.

**Table 2:** Experimental and theoretical values of glass transition temperatures of GTA- and TEC-plasticized cellulose acetate as determined by DSC measurements (second heating run).

Compound	CA/PL.-Ratio	$T_{\mathrm{ig}}$	$T_{\mathrm{mg}}$	$T_{\mathrm{fg}}$	$∆c_{p}$	$T_{g^{\mathrm{Comp}.}}^{F}{\;}^{2}$	$T_{g^{\mathrm{Comp}.}}^{\mathrm{KB}}{\;}^{2}$	$T_{g^{\mathrm{Comp}.}}^{\mathrm{CK}}{\;}^{2}$
	[wt%]	[°C]	[°C]	[°C]	[J g^−1^ K^−1^]	[°C]	[°C]	[°C]
CA	100	191.4	196.6	201.6	0.233	-	-	-
CA/GTA	85/15	109.6	122.8	137.1	0.143	120.2	112.0	98.4
	80/20	108.1	130.7	140.3	0.072	100.0	90.9	76.1
	75/25	86.3	104.1	122.6	0.150	81.8	72.2	57.1
	70/30	87.4	102.4	115.8	0.107	65.2	55.5	40.7
	65/35	63.4	85.7	92.6	0.063	50.1	40.7	26.4
	60/40	73.7	84.7	93.4	0.114	36.4	27.3	13.8
GTA **^1^**	100	−70.2	−68.4	−66.3	0.777	-	-	-
CA/TEC	85/15	129.5	142.4	153.4	0.157	118.8	109.5	88.9
	80/20	111.2	124.0	135.1	0.141	98.3	87.9	65.9
	75/25	96.5	115.6	128.7	0.121	79.9	69.0	46.8
	70/30	91.4	108.3	121.1	0.193	63.2	52.2	30.5
	65/35	90.1	100.0	113.2	0.071	48.0	37.2	16.6
	60/40	69.7	75.8	111.1	0.151	34.1	23.8	4.5
TEC **^1^**	100	−74.1	−70.9	−67.8	0.890	-	-	-

$T_{\mathrm{ig}}$—onset temperature; $T_{\mathrm{mg}}$—mid temperature; $T_{\mathrm{fg}}$—end temperature; $∆c_{p}$—specific heat capacity; $T_{g^{\mathrm{Comp}.}}^{F}$—theoretical glass transition temperatures of the compounds according to the Fox model; $T_{g^{\mathrm{Comp}.}}^{\mathrm{KB}}$—theoretical glass transition temperatures of the compounds according to the Kelley–Bueche model; $T_{g^{\mathrm{Comp}.}}^{\mathrm{CK}}$—theoretical glass transition temperatures of the compounds according to the Couchman–Karasz model. ^1^—transitions of the plasticizers (first heating run). ^2^—theoretical values of the glass transition temperatures (calculated with the prior experimentally determined values of the materials, not with the values from the technical datasheets).

**Table 3:** Relaxation temperatures and activation energies of the different plasticized cellulose acetate compounds determined from the maximum peak temperature of tanδ.

Compound	CA/PL.-Ratio	$T_{\alpha }^{\tan\delta }{\;}^{1}$	$T_{\beta }^{\tan\delta }{\;}^{1}$	$T_{\gamma }^{\tan\delta }{\;}^{1}$	$E_{a}^{\beta (\tan\delta )}$	$E_{a}^{\gamma (\tan\delta )}$
	[wt%]	[°C]	[°C]	[°C]	[kJ mol^−1^]	[kJ mol^−1^]
CA/GTA	85/15	145	−32	−104	92	23
	80/20	133	−25	−102	123	15
	75/25	114	−23	−101	144	19
	70/30	99	−24	−101	119	-
	65/35	83	−26	−102	167	-
	60/40	71	−29	-	153	-
CA/TEC	85/15	149	−30	−110	94	17
	80/20	140	−24	−104	118	22
	75/25	129	−21	−112	115	15
	70/30	121	−20	−109	131	16
	65/35	115	−20	−110	141	15
	60/40	94	−20	−106	140	15

$T_{\alpha }^{\tan\delta }—\alpha$-relaxation temperature assessed at the peak max. of tanδ; $T_{\beta }^{\tan\delta }—\beta$-relaxation temperature assessed at the peak max. of tanδ; $T_{\gamma }^{\tan\delta }—\gamma$-relaxation temperature assessed at the peak max. of tanδ; $E_{a}^{\beta (\tan\delta )}$—activation energies of the β-relaxation calculated from the peak max. of tanδ; $E_{a}^{\gamma (\tan\delta )}$—activation energies of the γ-relaxation calculated from the peak max. of tanδ.^1^—Relaxation temperatures assessed at a frequency of 1 Hz.

**Figure A1:**
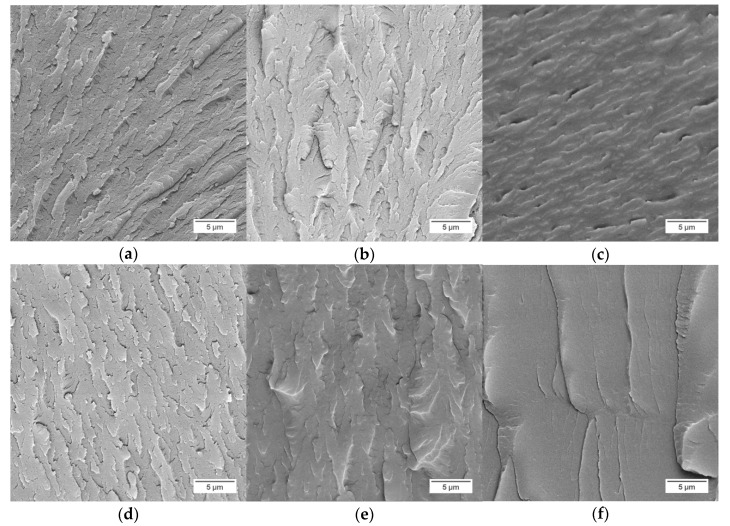
Cryo fracture surface of GTA- and TEC-plasticized cellulose acetate (**a**) 15 wt% GTA, (**b**) 25 wt% GTA, (**c**) 35 wt% GTA, (**d**) 15 wt% TEC, (**e**) 25 wt% TEC and (**f**) 35 wt% TEC.
